# A personal journey through academia as a neurodiverse scientist

**DOI:** 10.1038/s44319-025-00648-6

**Published:** 2025-11-24

**Authors:** Shina Caroline Lynn Kamerlin

**Affiliations:** Georgia Institute of Technology, School of Chemistry and Biochemistry, Atlanta, GA USA

**Keywords:** Careers

## Abstract

While neurodiversity is common in academia, it poses specific challenges for scientists. This piece shares my personal experience, as well as practical coping mechanisms for issues such as managing focus, handling anxiety spirals and dealing with social anxiety.

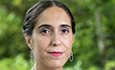

I recently participated in a collection of interviews for an excellent piece by Julian Nowogrodzki, who presented scientists with ADHD and how we navigate the challenges of academia (Nowogrodzki, 2025). Given that we usually do not talk about how we cope as neurodiverse researchers in academia—although this is fortunately changing—it was eye-opening to read about others who face similar challenges. I also received positive comments from colleagues who had seen the piece and recognized themselves in it. I therefore wanted to expand a bit more on my own experiences and coping strategies. While of course each of us has their individual challenges, hopefully you will find some of my solutions apply to your own journey.

To provide a bit of background, I grew up as a child in the 1980s and my ADHD and autistic traits were completely ignored; in fact, it took me several decades to even understand myself. For instance, I could obsessively listen to the same song over and over literally for hours, and I can rarely remember anyone’s name until I know them very well, or birth dates at all, or that I have horrible episodic memory, or that I am deeply anxious about social interactions, or my glaring issues with paying attention to anything that does not deeply interest me. Close friends have been upset with me because I cannot remember things we did together or their birthdays. And academia may not be the easiest career choice for someone with social anxiety, because, well, so much of our job depends on being social in various contexts.

Given the constraints of space, I wanted to focus here on four specific aspects of navigating academic life that I have found particularly challenging: issues with focus and procrastination; panic when overwhelmed by simultaneous deadlines; handling anxiety spirals; and navigating social situations.

While hyperfocus is my “superpower” and why I manage to get so much done once I actually get into things, the path to becoming focused is nothing if not bumpy. The “let me check this one other thing and suddenly two hours later…” stereotype is true for me and, has in fact, happened several times during the writing of this article. There are not only the distractions of both conventional and social media, but, characteristically of academia, the constant competing demands for your time and attention, and the constant distractions—whether it is an urgent email or someone who needs your input immediately for an urgent matter. It can be really hard to find focus and stay in it, and each time it’s broken, it can be hard to get back. I have ultimately succeeded to some extent by nannying myself as much as possible, first through apps that monitor my web browsing and block distractions, and then through self-discipline by removing distractions from my work laptop, and trying to control the frequency with which I check email. This has taken years of practice, and it does not mean I do not still procrastinate, but I have incrementally been able to force discipline on myself. Also, I have found it comforting to know that many of my senior colleagues battle the same problem, and I am not the only one who is unfocused.

If you have many things that need doing at once, common sense would be to prioritize and complete your tasks. Another characteristic of academia, however—one that only escalates the more senior you get—is the constant simultaneous demands on your time to complete things of maximum priority, often with identical or similar deadlines. There was a time when my reaction to this was truly debilitating. I would have a panic attack over how many things I have to do, paradoxically resulting in getting nothing done at all, thus further feeding into an anxiety spiral and aggravating the situation. It has taken a long time for me to learn to step back, snap out of it, and go for the low-hanging fruit and work my way up from there, one task at a time. This, in general, is how I solve any seemingly overwhelming problem, whether it is a personal, professional, or scientific problem: to deconstruct it and start with the easiest parts and work your way up. This also applies to exams: I advise my students to go for the easy points first and come back to harder questions like reverse peeling of an onion. That way you also feel both good about what you have done, and less demoralized by what is still ahead of you.

The panic attacks over being overwhelmed with deadlines are part of a more general tendency to get worried about things that may be genuinely serious, which can spiral over to things that are in hindsight extremely mundane. The first step to addressing an anxiety spiral is realizing you are in a spiral, and remembering that you might not be the most rational person in the world at that moment. If I am alone, I control my breathing, use a stress ball or something similar to squeeze, even wring my hands– anything that can help me take a step back. If you have someone you trust, the most helpful thing is to talk to that person, who can help to pull you back from the edge. This may seem tangential to the topic of navigating academia, but it’s actually not, given that how academia is set up frequently gives us things to spiral about.

The point I wish I could give a better answer to is how to handle social situations. I get deeply anxious when I am in a situation with a lot of people I do not know. I also have congenital ataxia due to an endocrine disorder, which means I am prone to poor coordination and clumsiness, which amplifies my overall social anxiety. I have recently found that, again, having a worry stone, stress ball, or even tugging at my bracelet helps a lot. If you are in a group, let other people do the talking, and get to know them first before you engage in the conversation. I am also open about the fact that I can’t remember people’s names or other things I may have forgotten due to my problems with episodic memory, and emphasize that it is not personal.

There are obviously positive aspects that help me a lot in science, including hyperfocus. In my case, it apparently is an ability to easily see abstract concepts and make connections and patterns in data. You will have your own individual strengths, and you should embrace and lean into them.

In addition to the interview, another reason I wanted to write this piece was a recent post on Quora, asking whether it is possible for someone with ADHD to be successful as a scientist, and seeing (unexpectedly) my name mentioned among the various examples. I hope that your take-home message from reading this column is that the answer to that question is a very resounding ‘yes!’, while also acknowledging and validating that the path to success has its own roadblocks, along with coping strategies you may find useful.
